# The multifaceted functions of β-arrestins and their therapeutic potential in neurodegenerative diseases

**DOI:** 10.1038/s12276-023-01144-4

**Published:** 2024-01-11

**Authors:** Teresa R. Kee, Sophia A. Khan, Maya B. Neidhart, Brianna M. Masters, Victoria K. Zhao, Yenna K. Kim, Kyle C. McGill Percy, Jung-A A. Woo

**Affiliations:** 1 Department of Pathology, CWRU School of Medicine, Cleveland, OH 44106 USA; 2 Department of Molecular Medicine, USF Health College of Medicine, Tampa, FL 33613 USA

**Keywords:** Neurodegeneration, Alzheimer's disease, Parkinson's disease

## Abstract

Arrestins are multifunctional proteins that regulate G-protein-coupled receptor (GPCR) desensitization, signaling, and internalization. The arrestin family consists of four subtypes: visual arrestin1, β-arrestin1, β-arrestin2, and visual arrestin-4. Recent studies have revealed the multifunctional roles of β-arrestins beyond GPCR signaling, including scaffolding and adapter functions, and physically interacting with non-GPCR receptors. Increasing evidence suggests that β-arrestins are involved in the pathogenesis of a variety of neurodegenerative diseases, including Alzheimer’s disease (AD), frontotemporal dementia (FTD), and Parkinson’s disease (PD). β-arrestins physically interact with γ-secretase, leading to increased production and accumulation of amyloid-beta in AD. Furthermore, β-arrestin oligomers inhibit the autophagy cargo receptor p62/SQSTM1, resulting in tau accumulation and aggregation in FTD. In PD, β-arrestins are upregulated in postmortem brain tissue and an MPTP model, and the β2AR regulates *SNCA* gene expression. In this review, we aim to provide an overview of β-arrestin1 and β-arrestin2, and describe their physiological functions and roles in neurodegenerative diseases. The multifaceted roles of β-arrestins and their involvement in neurodegenerative diseases suggest that they may serve as promising therapeutic targets.

## β-arrestins: unveiling their crucial role in neurodegenerative diseases

Alzheimer’s disease, Parkinson’s disease, frontotemporal dementia, and amyotrophic lateral sclerosis are neurodegenerative diseases impacting millions worldwide. Recent research has identified a significant association between these diseases and beta-arrestin, a regulatory protein that interacts with G-protein-coupled receptors (GPCRs). GPCRs play crucial roles in numerous biological processes and are the focus of many drug therapies. This review article provides a comprehensive summary of the established functions of beta-arrestins and underscores recent findings linking beta-arrestins to neurodegenerative diseases. Furthermore, it explores novel therapeutic avenues by targeting beta-arrestins in a manner that avoids disrupting their vital interactions with GPCRs. Researchers believe this strategy could lead to more efficient and specific treatments for these debilitating diseases.

## Introduction

### β-arrestin1 versus β-arrestin2

#### Expression patterns

β-arrestin1 and β-arrestin2 share 78% sequence similarity and thus share many functions, including regulating receptor desensitization^1,2^, internalization^3–6^, and signaling^1,7,8^. Double knockout of both β-arrestin1 and β-arrestin2 results in neonatal death^9^, while knockout of only one isoform results in a relatively normal phenotype^10,11^, indicating that one β-arrestin can functionally compensate for the loss of the other. β-arrestin1 and β-arrestin2 are ubiquitously expressed in most mammalian cell types and tissues^12,13^, including but not limited to epithelial^14,15^, endothelial^16–18^, and smooth muscle cells^19^. β-arrestins are also highly expressed in the brain^20^. Overall, β-arrestin1 is more abundant in the adult brain than β-arrestin2, with as much as a tenfold expression difference^21^, and the immunodensity of β-arrestin2 in the human brain decreases by 3–5% per decade^22^. However, expression patterns and cellular functions may vary depending on the specific brain region. For example, β-arrestin1 and β-arrestin2 have equal expression patterns in striatal medium spiny neuron (MSN) populations in both the direct and indirect dopamine pathways. Within the striatum, β-arrestin2 expression is higher in cholinergic interneurons than in parvalbumin interneurons or MSNs^23^. The prefrontal cortex exhibits higher β-arrestin2 expression than the striatum, whereas the levels of β-arrestin1 are similar between the two regions in the rat brain^24^. β-arrestin1 and β-arrestin2 are highly expressed in the pyramidal cell layers of the cingulate, somatosensory, and motor cortex, as well as in the CA1–CA3 regions and granular layer of the hippocampus and dentate gyrus (DG). β-arrestin1 also plays a role in neurogenesis and the proliferation of neural precursor cells in the subgranular zone and DG of the adult hippocampus^25^. Moderate to high expression is found in the hypothalamus and thalamic nuclei. Moderate expression is found in the periaqueductal gray, mesencephalic trigeminal nucleus, and locus coeruleus of the brainstem^20^.

#### Intracellular localization

β-arrestin1 is found in both the cytoplasm and nucleus, whereas β-arrestin2 is mainly found in the cytoplasm^26–28^. This differential cellular location is due to localization signal sequences. Both β-arrestin1 and β-arrestin2 contain a nuclear localization signal (NLS) in their N-terminus, which is required for nuclear import^26,28^. However, only β-arrestin2 contains a C-terminal leucine residue nuclear export signal (NES), which allows β-arrestin2 to exit the nucleus. Given its N-terminal NLS and C-terminal NES, β-arrestin2 shuttles between the cytoplasm and nucleus. Although β-arrestin2 is able to enter, its NES predominates and does not allow it to persist in the nucleus. This shuttling activity is important for the regulation of the E3 ubiquitin ligase Mdm2 and the protein kinase JNK3, which in turn regulate transcription factor activity^29^. Free cytoplasmic β-arrestins translocate to the plasma membrane upon agonist-induced receptor stimulation and/or act as scaffolding proteins^30^.

#### Differential interaction with GPCRs

β-arrestins interact with hundreds of GPCRs and participate in various signaling pathways to carry out their diverse cellular functions. These GPCRs include but are not limited to adrenergic, dopaminergic, opioid, cholinergic, and glutaminergic receptors. Many of these receptor-β-arrestin interactions are preferential for specific isoforms via biased agonist/ligand-induced recruitment. On a structural level, selective binding to receptors is indirectly mediated by the C-terminal region, directly mediated by the N-terminal region, and can also be mediated by the central domains of the β-arrestin protein^31^. GPCRs are categorized into six classes (A through F) according to functional and sequence similarities^27,32–34^. Currently, Class A GPCRs, also known as the “rhodopsin-like family”, are the largest and most diverse group of GPCRs^35^. The remaining classes, B through F, are classified as the secretin family, metabotropic glutamate receptors, fungal mating pheromone receptors, cyclic adenosine monophosphate receptors, and Frizzled and Smoothened receptors^32–34^. Generally, many Class A GPCRs tend to display a preference for β-arrestin2 over β-arrestin1, whereas Class B GPCRs lack such selectivity^27^. For example, it is well documented that β-arrestin2 has a dramatically greater affinity for beta-2 adrenergic receptor (β2AR) binding than β-arrestin1^36,37^ and translocates to the β2AR much more efficiently^27^. Similarly, evidence supports differential affinity between β-arrestin1 and β-arrestin2 with dopamine receptor isoforms. β-arrestins participate in dopaminergic signaling via interaction with dopamine 1 and dopamine 2 receptors (D1R and D2R). This protein‒receptor interaction occurs at the third cytoplasmic loop and C-terminal domain of the D1R and D2R. Given the structural similarities between β-arrestin1 and β-arrestin2, and between D1R and D2R, both isoforms of β-arrestin can interact with both isoforms of the dopamine receptor. However, β-arrestin1 and β-arrestin2 display agonist-induced preferential interactions with D2R^38^ and D1R^39^, respectively, in rodent striatal neurons. This preference infers a functional difference given that D1R typically complexes with Gs to stimulate adenylyl cyclase activity, while D2R typically complexes with Gi to inhibit adenylyl cyclase activity.

Among the opioid receptors, both β-arrestin isoforms interact with the Class A mu-opioid receptor (mOR) and regulate its diffusion and mobility. β-arrestin1 has been implicated in facilitating mOR dephosphorylation and potentially promoting its resensitization by mediating its ubiquitination. In contrast, β-arrestin2 is prominently involved in promoting receptor desensitization by recruiting various signaling proteins and promoting receptor internalization^40–45^. However, β-arrestin1 plays a greater role in mOR internalization than β-arrestin2, and β-arrestin1 knockdown suppresses, whereas β-arrestin2 knockdown increases, adenylyl cyclase activity^46^. Similarly, the delta-opioid receptor (dOR) engages in differential interactions with β-arrestins, as high-internalizing dOR agonists facilitate β-arrestin1 recruitment, whereas low-internalizing dOR agonists facilitate β-arrestin2 recruitment^47^. The dOR is also involved in the nuclear localization of β-arrestin1, as agonist-induced activation of dOR leads to the translocation of β-arrestin1 to the nucleus, where it can then stimulate transcription and histone acetylation^48^. This same induction of β-arrestin1 nuclear trafficking is not observed in agonist-stimulated mOR or β2AR.

Some Class B GPCRs known to interact with β-arrestins include, but are not limited to, the angiotensin II type 1A receptor (AT1aR), the muscarinic acetylcholine receptor (mAChR), and the parathyroid hormone 1 receptor (PTH1R). β-arrestin1 and β-arrestin2 are equally recruited to AT1aR in response to ligand activation. However, β-arrestin2 has the ability to induce a much greater affinity shift for certain biased agonists than β-arrestin1. Thus, although the two isoforms interact with AT1aR, their interactions may have different functions^49^. β-arrestin1 and β-arrestin2 are involved in the arrestin-dependent internalization of the M2 mAChR. While knockout of both β-arrestins in mouse embryonic fibroblasts dramatically diminishes mAChR internalization, the presence of either β-arrestin isoform can rescue mAChR internalization, indicating a lack of selectivity for either isoform^50^. Both β-arrestin1 and β-arrestin2 are recruited to the active PTH1R; however, small structural or conformational differences in the isoforms likely contribute to differential interactions with PTH1R conformations. β-arrestin1 preferably binds to and stabilizes the “hanging” PTH1R conformation to promote receptor internalization via interaction with distal receptor phosphorylation sites, whereas β-arrestin2 is unable to do so. Rather, β-arrestin2 preferably stabilizes the “core” PTH1R conformation via interaction with proximal receptor phosphorylation sites^51^. A summary of the similarities and differences between β-arrestin1 and β-arrestin2 can be found in Table 1.

**Table 1:** Comparison of β-arrestin1 and β-arrestin2.

	β-arrestin1	β-arrestin1 and β-arrestin2	β-arrestin2
Structure	Does not contain a C-terminal NES.	Share 78% sequence similarity^1,2^. Contain an N-terminal NLS for nuclear import^26,28^.	Contains a C-terminal NES, allowing for nuclear-cytoplasmic shuttling^29^.
Expression patterns	Up to 10× more abundant in the brain than β-arrestin2^21^.	Universally expressed in most mammalian cell types and tissues, and highly expressed in the brain^12,13,20^.	
	Protein concentration increases with age in the rat brain^21^.	Expression patterns are influenced by age.	Protein concentration decreases with age in the rat brain^21^, with an average immunodensity decrease of 3–5% per decade in the human brain^22^.
	Similar expression in the prefrontal cortex and striatum in the rat brain^24^.	Equal expression in striatal MSNs of direct and indirect dopamine pathways^23^. High expression in the pyramidal cells of the cortex and hippocampus^20^.	Higher expression in the prefrontal cortex compared to striatum in the rat brain^24^.
Class A GPCR interactions		Highly involved in the regulation of Class A GPCR signaling while similarly recruited by Class B GPCRs^27^.	Greater affinity for class A GPCRs compared to that of β-arrestin1^27^.
	There is very little research on the β-arrestin1-β2AR interaction despite the known high affinity between β-arrestin2 and the β2AR.	Most β2AR ligands trigger a similar degree of β-arrestin recruitment to the β2AR^180^.	Tenfold more efficient at internalizing the β2AR, and 100-fold greater amount of β-arrestin1 is needed to match β-arrestin2-driven β2AR internalization^37^. Significantly higher binding affinity for the β2AR than β-arrestin1^36,37^.
	Preferentially interacts with the D2R dopamine receptor isoform^38^.	Interact with D1R and D2R to participate in dopaminergic signaling^38,39^.	Preferentially interacts with the D1R dopamine receptor isoform^39^.
	Knockdown suppresses adenylyl cyclase activity^46^ High internalizing dOR agonists facilitate β-arrestin1 recruitment to the dOR^47^. β-arrestin1 plays a greater role in mOR internalization compared to β-arrestin2^46^.	Interact with opioid family receptors (mOR and dOR) and play a potentially neuroprotective role^181^.	Knockdown enhances adenylyl cyclase activity^46^ Low-internalizing dOR agonists facilitate β-arrestin2 recruitment to the dOR^47^.
Class B GPCR interactions		Equal interaction with the AT1aR with equal recruitment and binding affinity, albeit with slightly different conformations^49^.	Causes a greater affinity shift in response to certain agonists compared to β-arrestin1^49^.
	Preference for binding to and stabilizing the “hanging” PTH1R conformation^51^.	Equally recruited to the mAChR in response to ligand activation^50^ Equally recruited to the active PTH1R^51^.	Preference for binding to and stabilizing the “core” PTH1R conformation^51^.

## Physiological functions of β-arrestins

### β-arrestins in GPCR signaling

**Fig. 1:**
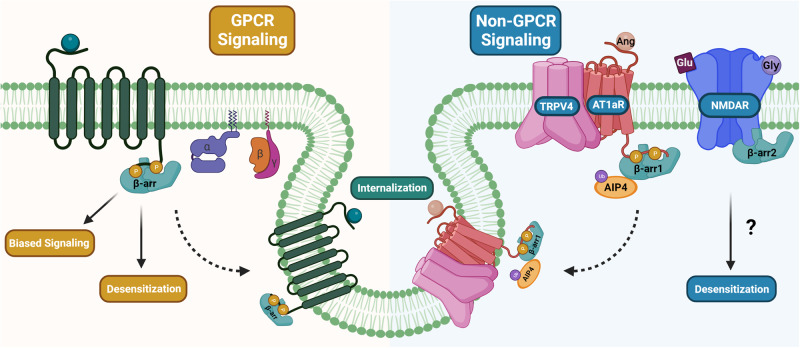
Physiological functions of β-arrestins. β-arrestins bind to the phosphorylated C-terminus of GPCRs, leading to desensitization, internalization, and β-arrestin-biased signaling. Upon binding to GPCRs, β-arrestin inhibits G-protein coupling, thereby desensitizing the receptor and promoting clathrin-mediated internalization. Additionally, β-arrestins perform inhibitory functions outside of GPCR binding. β-arrestin1 serves as an adapter protein by recruiting the E3 ubiquitin ligase AIP4 to the TRPV4 and AT1aR complex, resulting in internalization of the complex. β-arrestin2 is also involved in suppressing spinal cord NMDAR signaling. However, the precise mechanism by which β-arrestin2 downregulates spinal cord NMDAR signaling has yet to be fully elucidated. Created with BioRender.com.

β-arrestin1 and β-arrestin2 play a significant role in regulating GPCR function^52^ (Fig. 1). GPCRs interact with three main regulatory proteins: guanine nucleotide-binding proteins (G-proteins), G-protein-coupled receptor kinases (GRKs), and arrestins^53^. GPCR agonists activate heterotrimeric G-proteins by inducing a conformational change in the receptor. Upon activation, the α and βγ subunits of the G-protein dissociate, triggering the activation of secondary messenger-generating enzymes. As a result, the cell may undergo a variety of physiological changes^53^. However, there is a system that immediately opposes the effects of G-protein activation, beginning with the phosphorylation of the activated receptor by a GRK^52^. This first step sequentially leads to β-arrestin binding to the phosphorylated receptor, as receptor phosphorylation increases the binding affinity between β-arrestin and the receptor. G-protein coupling is physically inhibited by β-arrestin binding, resulting in homologous desensitization of the receptor^52^ (Fig. 1). Overall, this is an established paradigm of the mechanisms of GPCRs, which was discovered when studying the β2AR^54^.

Along with desensitization, the binding of β-arrestins to GRK-phosphorylated receptors also facilitates GPCR sequestration or internalization (Fig. 1). β-arrestins can function as adapter proteins because they contain two motifs in the R2 domain of their C-terminus: the clathrin binding motif LIEF and AP-2 binding motif RXR^52,55^. These motifs allow them to create a linkage between the GPCR and clathrin-dependent endocytic machinery^52^. The interaction between the β2 adaptin subunit of AP-2 recruits activated β-arrestins to the clathrin pit via the RXR motif, which is found in the autoinhibitory C-terminus of all arrestin subtypes^56–58^. However, interactions between clathrin and β-arrestins can also occur due to the presence of the LIEF motif within the autoinhibitory segment of β-arrestin1 and β-arrestin2^4,57,59^. The inactive C-terminal ‘tail’ of β-arrestin acts as an autoinhibitory segment bound to a groove in the N-terminus^14,57^ and, as a result, masks AP-2 and clathrin binding motifs. When the C-terminus is phosphorylated, however, it will displace this autoinhibitory tail segment, allowing for interaction between β-arrestin, AP-2, and clathrin. This interaction results in the recruitment of GPCRs to clathrin-coated pits^57,60,61^.

β-arrestin1 is basally phosphorylated by ERK1 and ERK2 at Ser-412 and is dephosphorylated at the plasma membrane prior to receptor binding^62,63^. This finding was confirmed by the expression of a dominant-negative MEK1 mutant (K97A) in HEK293 cells, resulting in *a*~70% reduction in phosphorylated β-arrestin1^62^. β-arrestin1 dephosphorylation is required for binding to clathrin and internalization of β2AR^63^, and MEK1 K97A increases this interaction, leading to receptor sequestration by increasing dephosphorylated β-arrestin1^62^. Expression of a constitutively active MEK1 S218D/S222D mutant reduces receptor sequestration, which is enhanced by overexpression of β-arrestin1^62^. However, overexpression of β-arrestin2 with MEK1 S218D/S222D has no effect on receptor sequestration^62^, suggesting that ERKs only modulate the function of β-arrestin1 and not β-arrestin2, as β-arrestin2 does not have a comparable Ser-412 site.

β-arrestin2 also functions as an adapter protein and recruits Mdm2, an E3 ubiquitin ligase that ubiquitinates both β2AR and β-arrestin2^64^. Loss or inactivation of Mdm2 inhibits the internalization, but not degradation, of β2AR^64^, indicating that the ubiquitination of β-arrestin2 is required for proper receptor internalization. Furthermore, the expression of a β2AR mutant without lysine residues, which prevents ubiquitination, led to improper degradation of the receptor^64^. These results indicate that β-arrestin2 and GPCR ubiquitination play distinct roles in receptor internalization and degradation. The β2AR can be catalytically ubiquitinated by other unidentified E3 ligases, but this still requires the presence of β-arrestin2 for proper internalization^4,64^. This is because β-arrestin2 quickly binds in response to accumulated GRK2-mediated phosphorylated receptors and immediately dissociates upon agonist removal^65^. This fast-acting mechanism of β-arrestin2 is necessary for rapid signaling control in cells such as neurons. Clathrin and AP-2 are two integral proteins of clathrin-coated pits where GPCR internalization occurs^4^.

### β-arrestins in biased signaling

As previously described, classic GPCR signaling involves agonist binding to the receptor, leading to the activation of heterotrimeric G-proteins and downstream second messenger signaling. After phosphorylation of the receptor C-terminus by GRKs, β-arrestins are recruited and bind to the receptor, leading to the steric hindrance of further G-protein signaling. However, β-arrestin proteins can simultaneously trigger another separate set of downstream signals^66,67^ (Fig. 1). This is known as “β-arrestin-biased signaling.” Agonist testing has determined that certain ligands may be biased toward G-protein and/or β-arrestin signaling, leading to different downstream signaling processes^66,67^. Additionally, β-arrestin-biased ligands may simultaneously inhibit G-protein signaling while promoting β-arrestin signaling^66,67^. This concept of receptor bias could be used to prevent the off-target effects of certain drugs, therefore leading to a reduction in side effects.

While many different receptor types have been found to be capable of biased signaling, one of the most studied are β-adrenergic receptors (β-ARs). Indeed, most known agonists of β2AR exhibit β-arrestin bias and demonstrate similar G-protein signaling activity^68,69^. Three β2AR agonists show clear β-arrestin bias: isoetharane, clenbuterol, and ethylnorepinephrine^68^. Interestingly, all three biased agonists contain an ethyl substitution on the α-carbon^68^, indicating that biased ligands may have similar structures. Further investigation into the biased ligands of β-ARs has revealed an important role of β-arrestin-biased signaling in cognitive functions. β-arrestin-biased signaling through the β-adrenergic signaling pathway is required for memory reconsolidation^70^, suggesting a novel target for drug therapies.

β-arrestin-biased signaling has also been observed in dopamine receptors. Novel D2R agonists that are β-arrestin biased can exert antipsychotic effects without off-target motor side effects^71^. This finding was confirmed by a lack of response in β-arrestin2 knockout mice^71^. Moreover, structural studies may be able to determine which compounds are able to induce β-arrestin-biased activity^72^. Further testing of a β-arrestin-biased D2R ligand showed promise in the treatment of schizophrenia by acting as both an agonist and antagonist in different brain regions, leading to a broader effect on symptoms^24^. These data support the use of β-arrestin-biased agonists for more specific pharmacological targeting, as they may be able to reduce side effects and increase effectiveness.

### β-arrestins in non-GPCR signaling

β-arrestins are multifunctional proteins that regulate various non-GPCR signaling pathways^73^. β-arrestin1 plays a role in focal adhesion kinase (FAK) activation via C-X-C chemokine receptor type 4 (CXCR4), a GPCR, resulting in chemotaxis^74^. This interaction is facilitated by STAM1 binding to β-arrestin1, thereby creating a structural conformation specific for CXCR4 activation^74^. These data highlight a mechanism by which the non-GPCR functions of β-arrestin1 may contribute to the downstream regulation of GPCRs. β-arrestin1 also acts as an adapter in the regulation of TRPV4, a mechanosensitive Ca^2+^ channel. β-arrestin1 is recruited to TRPV4 and the AT1aR complex via angiotensin stimulation^75^ (Fig. 1). β-arrestin1 then acts as an adapter for the E3 ubiquitin ligase AIP4, which ubiquitinates TRPV4, resulting in downregulation of the ion channel^75^. This pathway serves as an excellent example of the critical function β-arrestins play as adapters, recruiting E3 ligases and subsequently facilitating the ubiquitination of adjacent receptors^76^. β-arrestin2 also regulates TRPV1, a cation channel in the transient receptor potential family. β-arrestin2 forms a complex with the phosphodiesterase PDE4D5, resulting in the desensitization of TRPV1^77^. These findings further indicate that the scaffolding properties of both β-arrestins play a significant regulatory role outside of the scope of GPCRs.

β-arrestin2 also interacts with N-methyl-D-aspartate receptors (NMDARs) in the spinal cord dorsal horn^78^ (Fig. 1). NMDARs are glutamate-sensitive ion channels composed of GluN1 and GluN2 or GluN3^79^. NMDARs mainly function at excitatory synapses, regulating sodium and calcium influx and potassium efflux in the central nervous system^80^. Their activation requires glutamate and either glycine or D-serine binding^80^. β-arrestin2 knockout mice display prolonged GluN2B-dependent late-phase mechanical allodynia and early-phase analgesia^78^. In contrast, overexpression of β-arrestin2 resolves chronic neuropathic pain^78^. This finding suggests that β-arrestin2 may also serve as a regulator of spinal cord NMDARs associated with the duration of pain through the desensitization of NMDARs; however, the specific mechanism behind this signaling pathway is unclear, and it remains to be tested whether there is a direct interaction between β-arrestin2 and NMDARs^78^.

β-arrestin2 functions in multiple antiproliferative pathways by regulating the type III transforming growth factor beta (TGF-β) receptor and maternal embryonic leucine-zipper kinase (MELK). β-arrestin2 regulates TGF-β function via receptor internalization, resulting in decreased TGF-β signaling^81,82^. MELK plays a role in cell cycle regulation, proliferation, and apoptosis. Additionally, overexpression of MELK impacts various cancer types, such as glioblastomas^82^. The interaction between β-arrestin2 and MELK influences the function of the active MELK kinase domain, promoting the antiproliferative MELK pathway^82^. However, the exact nature of the interactions between β-arrestin2 and MELK has yet to be elucidated. β-arrestins also play a role in regulating transcription factors via Smoothened (Smo). β-arrestins play a role in Sonic Hedgehog (Shh) signaling through Smo, a GPCR. Both β-arrestin1 and β-arrestin2 mediate the interaction between Smo and Kif3A, a kinesin motor protein, allowing Smo to localize to the primary cilia and activate Gli^83^. This pathway is supported by a study showing that Smo localization to primary cilia in NIH3T3 cells is disrupted after transfection with β-arrestin1 and β-arrestin2 siRNA^83^. These studies indicate that β-arrestin2 plays a significant role in the regulation of proliferative pathways such as MELK and that both β-arrestins aid in the regulation of Shh, independent of GPCR binding.

β-arrestin1 and β-arrestin2 exist in monomer, homo- and hetero-oligomer forms^84,85^. β-arrestin oligomers are formed by bridging binding sites located on the N- and C-terminal globular domains, and binding is facilitated by inositol hexakisphosphate (IP6)^85^. β-arrestin oligomers have previously been proposed as an inactive, “resting” state; however, multiple studies have highlighted their unique functional roles. While β-arrestin monomers are mostly thought to act on GPCRs, β-arrestin2 oligomers have been shown to still bind to AT1aR after stimulation with the same kinetics as monomers^84^, suggesting that oligomers play functional roles. Disturbing the NES of β-arrestin2 results in the accumulation of both endogenous and mutant β-arrestin2 in the nucleus^84^. However, co-expression of β-arrestin1 and β-arrestin2 allows β-arrestin1 to be exported from the nucleus, suggesting that hetero-oligomers help maintain the correct subcellular localization of β-arrestins. A previous study showed that β-arrestin2 oligomers interact with the oncoprotein Mdm2 and promote its nuclear export, enhancing the antiproliferative effects of p53^86^. However, β-arrestin2 oligomerization mutants reduce their interaction with Mdm2, which inhibits p53 downstream effects^86^, suggesting that β-arrestin2 oligomers regulate cell proliferation and survival. β-arrestin1 and β-arrestin2 oligomers also disrupt autophagy machinery by preventing p62/SQSTM1 self-interaction, leading to the accumulation of pathological tau in FTD^87,88^. β-arrestin1 can also associate with microtubules and compete with tau for microtubule binding, leading to microtubule destabilization in FTD^88^. These results indicate that the differing conformations of β-arrestins allow them to participate in unique cellular functions.

## Role of β-arrestins in neurodegenerative diseases

### β-arrestins in Alzheimer’s disease

**Fig. 2:**
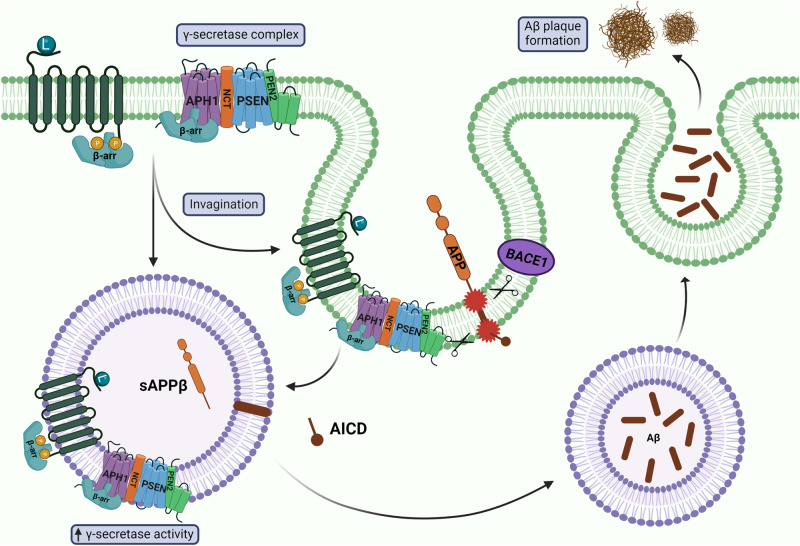
β-arrestins in Alzheimer’s disease. In AD, β-arrestins directly bind to the APH1 subunit of γ-secretase, which subsequently leads to an increase in γ-secretase enzymatic activity. Amyloid precursor protein (APP) is cleaved by γ-secretase and BACE1, leading to the production of toxic Aβ. The enhanced gamma-secretase activity within endosomes further contributes to the increased production of toxic Aβ peptides. Endosomes containing Aβ are exocytosed, leading to the formation of extracellular Aβ plaques. Created with BioRender.com.

Alzheimer’s disease (AD) is a progressive neurodegenerative disease characterized by the accumulation of toxic intracellular tau tangles and extracellular amyloid-beta (Aβ) plaques in the brain^89^. Interestingly, β-arrestin1 and β-arrestin2 mRNA and protein levels are elevated in postmortem brain tissues from AD patients^90–93^. Overexpression of β-arrestin1 or β-arrestin2 increases Aβ production in cultured cells and primary neurons in vitro, and genetic ablation of *Arrb1* or *Arrb2* reduces Aβ accumulation in the *APP/PS1* mouse model of AD^91,92^. Moreover, compared to their *APP/PS1* littermates, *APP/PS1;Arrb1*^−^^/^^−^ mice exhibit improved learning and memory in the novel object recognition and Morris water maze tests^91^. Furthermore, siRNA knockdown of β-arrestin1 in CRND8 mice, an APP mouse model with Swedish and Indiana mutations, significantly reduces the Aβ plaque burden in the hippocampus^91^. β-arrestin1 and β-arrestin2 modulate Aβ levels by directly interacting with the γ-secretase complex^91,92^ (Fig. 2). Specifically, β-arrestin1 and β-arrestin2 bind to the anterior pharynx defective 1 (APH1) subunit of the γ-secretase complex^91,92^, and disruption of this interaction reduces Aβ production in vitro^91^. It was recently revealed that APH1A contains GRK-mediated phosphorylation barcodes in its C-terminus, which regulate the recruitment and interaction with β-arrestin2^94^, thereby regulating Aβ levels.

APP cleavage, Aβ production, and γ-secretase activity can be altered via β2AR and G-protein-coupled receptor 3 (GPR3). β-arrestin2 has a higher binding affinity for both β2AR and GPR3 than β-arrestin1, and treatment with the β2AR agonist isoproterenol in APPswe HEK293 cells increases Aβ_40_ and Aβ_42_ levels^95^. However, cotreatment with the β2AR antagonists propranolol and isoproterenol eradicates the increase in secreted Aβ levels, while propranolol treatment alone has no effect^95^. Pretreatment of the γ-secretase inhibitor L895,458 alongside isoproterenol does not alter secreted Aβ^95^ levels, indicating that β2AR modulates Aβ levels in a γ-secretase-dependent manner. Furthermore, stimulation of β2AR increases the production of Aβ in late endosomes and lysosomes^95^. Isoproterenol treatment also enhances the colocalization of RAB7 and PS1 in endosomes^95^, and acidic environments enhance γ-secretase activity, leading to an increase in Aβ production^96^. Knockdown of β-arrestin2, but not β-arrestin1, prevents the internalization of β2AR after Aβ or isoproterenol treatment in mouse embryonic fibroblasts (MEFs)^97^. Additionally, β-arrestin2 V54D, a mutant incapable of inducing β2AR internalization, is also unresponsive to Aβ or isoproterenol treatment in HEK293 cells^97^, further suggesting that β2AR internalization is primarily reliant on β-arrestin2.

Additionally, GPR3 overexpression increases Aβ levels, and GPR3 knockdown reduces Aβ levels in vitro^98,99^. Inhibition of γ-secretase eliminates GPR3’s effect on Aβ production in HEK293 cells and mouse primary hippocampal neurons^98^. Transduction of GPR3 in the *APP/PS1* hippocampus increases Aβ levels without altering γ-secretase expression levels, and *APP/PS1;Gpr3*^*+/*^^−^ and *APP/PS1;Gpr3*^−^^*/*^^−^ mice exhibit significantly reduced Aβ levels compared to *APP/PS1* littermates. These results suggest that GPR3 is also dependent on γ-secretase for Aβ regulation; however, a direct interaction has not yet been observed. Co-transfection of GPR3 and β-arrestin2, but not β-arrestin1, significantly increases Aβ levels in vitro, and knockdown of β-arrestin2 suppresses GPR3-induced Aβ production^99^. Point mutations in GPR3 impair Gs coupling (DRY-AAY) and eliminate β-arrestin recruitment (Q302*), while a point mutation that removes a putative GRK site (S237A) enhances GPR3-β-arrestin2 interaction^99^. GPR3 Q302* also failed to increase Aβ^99^, suggesting that the GPR3-β-arrestin2 interaction is required to stimulate Aβ production. GPR3 colocalizes with APP in rat hippocampal neurons and with β-arrestin2 in neuronal cell bodies and endosomes^99^. Overexpression of β-arrestin2 or the GPR3 S237A variant significantly enhances, while DRY-AYY and Q302* variants reduce GPR3-APP interaction, and β-arrestin1 overexpression has no effect^99^. This finding correlates with β-arrestin2 recruitment to GPR3, suggesting that GPR3-β-arrestin2 binding is needed to enhance Aβ production. G-protein-biased GPR3 mice, which retain G-protein signaling while eradicating β-arrestin2 signaling, crossed with *APP* knock-in mice exhibit significantly reduced Aβ levels and neuroinflammation^100^, further supporting that β-arrestin2 signaling is required for GPR3-mediated Aβ production.

Neuroinflammation is another hallmark of AD, and GPCRs on microglia can promote or reduce Aβ pathology^101^. Aβ can directly bind to multiple GPCRs located on microglia and regulate APP processing^102^. Furthermore, treatment with various modulators of microglial GPCRs can result in a wide range of effects on neuroinflammation, Aβ pathology, and neurotoxicity^101^. GRKs have also been implicated in the pathogenesis of AD. GRK2 is upregulated in the brain in early-stage AD^103^, and in vitro Aβ treatment reduces membrane-associated GRK2 and GRK5, which promotes GPCR dysfunction^104^. In vivo studies revealed a similar reduction in active GRK2 and GRK5 levels in CRND8 AD mice^104^, and membrane-associated GRK5 levels are reduced, while cytosolic levels are increased, in aged APPswe mice^105^. Total GRK2 levels are increased in peripheral blood samples from AD patients^106^, and Aβ can induce tau phosphorylation at Ser-214 via a GRK2/β2AR/PKA signaling pathway^107^. These studies reveal key connections between Aβ, microglial GPCRs, and GRKs in the pathogenesis of AD. However, further research is needed on the specific role of β-arrestins in neuroinflammation and GRK dysfunction in the context of AD.

Current AD therapies include cholinesterase inhibitors, NMDA antagonists, and immunotherapies. Cholinesterase inhibitors such as donepezil, rivastigmine, and galantamine prevent the degradation of acetylcholine and butyrylcholine, thereby increasing levels in the brain^108^. Cholines can bind to multiple receptor types, including nicotinic and muscarinic acetylcholine receptors, in which the latter is a type of GPCR. NMDA antagonists such as memantine regulate glutamate signaling in the brain by inhibiting non-GPCR NMDA receptors^109^. Immunotherapies for the treatment of AD include recently FDA-approved aducanumab and lecanemab, which are Aβ monoclonal antibody treatments^110^. However, the role of β-arrestins in current AD therapies has not been explored, outside of their canonical role in regulating GPCR signaling.

### β-arrestins in frontotemporal dementia

Frontotemporal dementia (FTD) is an ill-defined group of neurodegenerative diseases largely characterized by frontotemporal lobar degeneration (FTLD)^111^. First described as Pick’s disease, FTD has been difficult to clinically define due to the wide range of presentations and pathologies. FTD differs from AD in both the brain regions that degenerate and the lack of Aβ pathology^112^. Despite challenges with clinically diagnosing FTD, there are currently two major classifications of FTD based on pathology: FTLD-tau and FTLD-TDP, which are characterized by tauopathy and TDP-43 proteinopathy, respectively^113^. Despite being a significant cause of dementia, FTD is not as well studied as AD, and the role of β-arrestins in FTD is poorly understood, with only two studies investigating β-arrestins in FTLD-tau. However, both β-arrestin1 and β-arrestin2 levels are elevated in FTLD-tau patients and PS19 transgenic mice, suggesting that β-arrestins may play a key role in FTLD-tau pathology^87,88^.

**Fig. 3:**
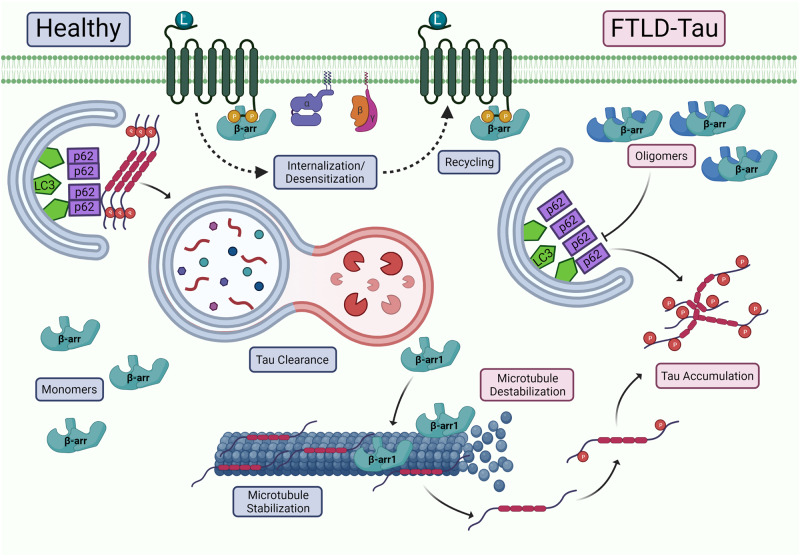
β-arrestins in frontotemporal dementia. In healthy neurons, β-arrestin monomers regulate the internalization, desensitization, and recycling of GPCRs. Additionally, P62/SQSTM1-mediated autophagy remains intact due to the successful self-interaction of P62, which is required for its localization to the autophagosome formation site, resulting in efficient removal of damaged organelles and misfolded proteins. Tau is able to bind to and stabilize microtubules. However, in FTLD-Tau, β-arrestin oligomers accumulate and prevent P62/SQSTM1 self-interaction, leading to autophagy impairment. β-arrestin1 also competes with tau for microtubule binding, inducing microtubule destabilization and tau accumulation. Created with BioRender.com.

β-arrestin2 overexpression increases, while β-arrestin2 knockdown decreases, tau and phospho-tau levels in HeLa-V5-tau cells and PS19 primary cortical neurons^87^. However, tau mRNA levels are not significantly altered, suggesting that β-arrestin2 affects tau levels at a post-translational level^87^. PS19;*Arrb2*^−^^/^^−^ mice have decreased sarkosyl-insoluble tau and phospho-tau, and hippocampal long-term potentiation is restored in PS19;*Arrb2*^+/^^−^ and PS19;*Arrb2*^−^^/^^−^ mice compared to PS19 littermates^87^. β-arrestin2 oligomeric mutants ΔIP6N and ΔIP6C increase tau turnover and autophagy flux in HeLa-V5-tau cells^87^. Furthermore, AAV9 expression of ΔIP6N and ΔIP6C mutants in PS19 mice inhibits hippocampal tau accumulation in vivo^87^. β-arrestin2 also inhibits p62/SQSTM1 self-interaction (Fig. 3), while ΔIP6N and ΔIP6C mutants do not, suggesting that β-arrestin2 oligomers specifically affect autophagy via p62/SQSTM1^87^. These results suggest that β-arrestin2 oligomers may be a therapeutic target for FTLD-tau or other tauopathies.

Similarly, β-arrestin1 is elevated in and colocalizes with pathogenic AT8 phospho-tau in FTLD-tau patient postmortem brain tissue, with no significant difference in mRNA levels^88^. β-arrestin1 and β-arrestin2 knockdown reduces GPCR agonist-induced increases in tau in HeLa-V5-tau cells, suggesting that β-arrestins are necessary for GPCR-mediated increases in tau and phospho-tau^88^. Overexpression of β-arrestin1 reduces the amount of tau bound to microtubules, suggesting that β-arrestin1 promotes tau missorting via dissociation from microtubules^88^ (Fig. 3). Furthermore, compared to PS19 littermates, PS19;*Arrb1*^−^^/^^−^ mice exhibit reduced tau accumulation and improved cognitive function^88^. Similar to β-arrestin2, β-arrestin1 inhibits p62/SQSTM1 flux by disrupting p62 self-association^88^ (Fig. 3). The effects of β-arrestin1 and β-arrestin2 on tauopathy warrant further investigation into their mechanisms and therapeutic potential for other tauopathies outside of FTD. While multiple studies have connected GRKs to AD pathology, the roles of GRKs in FTD are not well understood. FTD therapies include selective serotonin reuptake inhibitors and antipsychotics for the management of behavioral symptoms and riluzole and dopamine for motor symptoms^114^. While these therapies may assist in reducing symptomatic behaviors, there are currently no treatments that target the underlying cause of the disease. Indeed, GPCR dysfunction and neuroinflammation are also hallmarks of FTD; however, the specific roles of β-arrestins in the context of FTD need to be further explored in these areas.

### β-arrestins in Parkinson’s disease

Parkinson’s disease (PD) is the second most prevalent progressive neurodegenerative disorder that affects older adults after AD^115,116^. Its prevalence is expected to increase nearly exponentially as the population ages, with risk peaking after 80 years of age^115,117,118^. The main PD hallmarks are the loss of dopaminergic neurons in the substantia nigra of the midbrain and the accumulation of intracellular inclusions of α-synuclein protein known as Lewy bodies^115,119–121^. Motor symptoms of PD include bradykinesia, muscular rigidity, and resting tremors, and nonmotor symptoms include olfactory dysfunction, cognitive impairment, psychiatric symptoms, and autonomic dysfunction^119^. Currently available therapies only treat symptoms of the disease;^115,119^ thus, a major objective in PD research is the development of drugs that slow or halt the neurodegenerative process entirely.

**Fig. 4:**
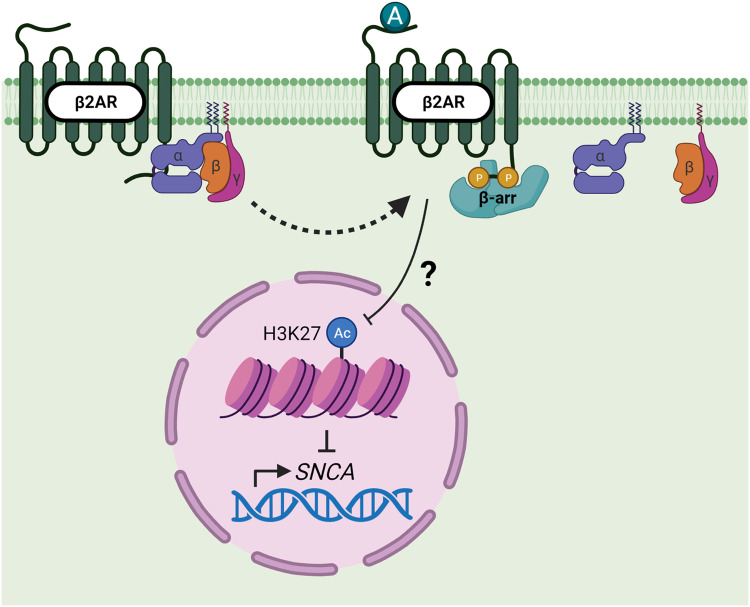
The β2AR regulates *SNCA* gene expression. Binding of β2AR agonists (A), such as clenbuterol, to the receptor induces the dissociation of heterotrimeric G-proteins, leading to receptor phosphorylation and β-arrestin binding. Although the precise molecular mechanism underlying the reduction in H3K27 acetylation after clenbuterol treatment remains unknown, it has been observed that this reduction ultimately leads to a reduction in *SNCA* gene expression. However, the role of β-arrestins in this process has not been investigated. Created with BioRender.com.

GPCR signaling is involved in PD pathogenesis, as GPCRs mediate microglial activation, which can be proinflammatory and neurotoxic when active, contributing to PD pathology^119,122–125^. β-arrestin1 and β-arrestin2 have opposing effects on neuroinflammation and microglia, as knockout of β-arrestin1 improves, while knockout of β-arrestin2 exacerbates, PD pathological features through microglial activation and dopaminergic neuron death^126^. These differing effects that β-arrestin1 and β-arrestin2 have on PD pathology may be due to their regulatory effects on the inflammatory NF-κB and STAT1 pathways. Knockout of β-arrestin1 inhibits components of the NF-κB and STAT1 pathways, while β-arrestin2 knockout activates the same signaling proteins, leading to reduced and increased inflammation, respectively^126^. To further interpret these differing effects, the expression levels of β-arrestin1 and β-arrestin2 across immune cells should be considered^19^. Both β-arrestins are expressed in macrophages^127^ and lymphocytes^128^. However, T cells express more β-arrestin2 than B cells^128^, and β-arrestin1 is highly expressed specifically in polymorphonuclear leukocytes^16^. β-arrestin expression levels across immune cells may vary in disease states. For example, typical CD4+ T lymphocytes highly express nuclear β-arrestin1; however, in allergic asthma mouse models, CD4+ T lymphocytes highly express β-arrestin2^129^. Specifics of β-arrestin expression patterns in proinflammatory and anti-inflammatory immune cells require further research. Additionally, α-synuclein interferes with GPCR signaling^130^, and β2AR regulates the expression of *SNCA* (Fig. 4), the gene that encodes α-synuclein^131^. Therefore, elucidating the relationship between GPCR signaling, α-synuclein, and β-arrestins is essential for further understanding PD pathology.

β-arrestins mediate dopamine receptor (DAR) signaling, which contributes to PD pathogenesis^132^. GRKs and β-arrestins are upregulated in PD models, although the upregulated GRK subtype varies^133,134^. For example, in a 1-methyl-4-phenyl-1,2,3,6-tetrahydropyridine (MPTP) PD model, β-arrestin1 and GRK6 are upregulated^133^. However, GRK3 and GRK5 are upregulated in human postmortem tissue from Parkinson’s disease with dementia (PDD) patients, while GRK6 expression is not altered. As both β-arrestin1 and β-arrestin2 demonstrate upregulated expression in PD^133,134^, it has been suggested that downregulating GRK and β-arrestin expression may serve as a therapeutic target. Post-MPTP lesioning, L-DOPA treatment reduces β-arrestin1 and GRK6 expression levels^133^. Simultaneous upregulation of GRKs and β-arrestins suggests that they may be expressed concurrently, although whether they colocalize has not been fully established. Interestingly, GRK2 and GRK5 can phosphorylate α-synuclein at the C-terminus, and phosphorylation is regulated by factors that also regulate GPCR phosphorylation^135^. However, the role of β-arrestins in α-synuclein phosphorylation by GRKs has not been investigated. While the interactions between GRKs, α-synuclein, and β-arrestins within PD pathogenesis have not been fully elucidated, it appears that they all contribute to DAR regulation and signaling, which is an essential aspect of PD pathogenesis.

Current antiparkinsonian therapies include dopamine precursors, dopamine agonists, and adenosine receptor antagonists. Undesirable side effects of these medications may be exacerbated or alleviated by targeting β-arrestins. Concurrently, antiparkinsonian compounds may affect β-arrestins’ involvement in PD progression. Thus, β-arrestins are a target of interest for maximizing the efficacy and reducing the side effects of PD therapies. L-DOPA or levodopa, a dopamine precursor, is a standard treatment for bradykinesia and parkinsonism in PD^136^. Piribedil, a D2R and D3R agonist, is another standard PD treatment typically used in conjunction with L-DOPA^137,138^. Another common complementary treatment, istradefylline, is an adenosine A2A receptor antagonist that is also used in combination with L-DOPA and is used to treat “off” periods between medications during which PD symptoms are heightened^139^. L-DOPA-induced dyskinesias (LIDs) are a side effect of L-DOPA use and are characterized by involuntary, excessive muscle movement^140,141^. After five to six months of treatment, approximately 50% of patients present with one or more LID, with a median prevalence of approximately 40% after four to six years of treatment, suggesting the need for LID-targeted therapies^140–142^. Dopaminergic presynaptic denervation in the nigrostriatal pathway, increased striatal DAR activity, and GPCR hypersensitivity contribute to LID pathogenesis^143–146^. Because of its role in desensitizing GPCR signaling, β-arrestin2 is a protein target of interest for alleviating LID symptoms^147,148^. Overexpression of β-arrestin2 reduces LID-associated symptoms, while genetic deletion of *Arrb2* increases LID-associated symptoms^148^. In a 6-hydroxydopamine-lesioned model of PD, β-arrestin2 overexpression attenuates LID-associated symptoms and decreases D1R activation and the levels of D1R-associated markers FosB, ERK1/2 phosphorylation, and DARPP-32^144,149^. However, β-arrestin2 overexpression does not affect the antiparkinsonian effects of L-DOPA. Conversely, knocking down β-arrestin2 promotes dyskinetic symptoms and increases ERK1/2 phosphorylation, FosB, and DARPP-32. These findings further support the notion that β-arrestin2 may mediate D1R activity by decreasing GPCR sensitivity and could be a possible therapeutic target for LIDs.

As previously mentioned, β-arrestins play a role in Aβ accumulation, which is a comorbid aspect of PD pathology associated with cognitive symptoms^137,150^. PD treatments L-DOPA, piribedil, and istradefylline can promote Aβ generation. For example, piribedil and L-DOPA stimulate Aβ production in primary neurons and neuronal cells^137^. It has been suggested that D2R expression and activation from β-arrestin signaling are primarily responsible for increased γ-secretase activity^137^. Accordingly, when β-arrestin2 is knocked down, Aβ production and γ-secretase activity are inhibited^137^. Istradefylline also promotes Aβ production in vitro, but this effect is not dependent on β-arrestins^151^. β-arrestin2 may have differential effects on the D1R and D2R signaling pathways, and further characterization of this relationship may elucidate more specific therapies for PD.

Autophagy dysfunction is another hallmark of PD and other neurodegenerative diseases^152–155^. Chaperone-mediated autophagy (CMA) can degrade α-synuclein, as inhibition of CMA induces the accumulation of soluble high molecular weight and detergent-insoluble species of α-synuclein^156^. Additionally, β-arrestin1 and β-arrestin2 oligomers inhibit autophagy flux by preventing p62/SQSTM1 self-interaction, leading to the accumulation of pathological tau in FTLD^87,88^. While this mechanism has not yet been explored in the context of PD, it is plausible that β-arrestin oligomers have a similar effect on α-synucleinopathy. Agonists of β2AR reduce, while antagonists increase, *SNCA* gene expression by modulating H3K27 acetylation^131^. In a population of 4 million Norwegians, treatment with β2AR agonists reduced, while antagonist treatment increased, the risk of developing PD^131^. Furthermore, tau is a common co-pathology seen in PD patients and patients with other Lewy body disorders^157–159^, and tau is required for memory and synaptic dysfunction in a mouse model of α-synucleinopathy^160^.

### β-arrestins in amyotrophic lateral sclerosis

Amyotrophic lateral sclerosis (ALS) is a neurodegenerative disease characterized by motor neuron degeneration and loss. Clinical symptoms include loss of voluntary muscle use, stiffness, dyspnea, wasting, dysarthria, and eventually paralysis^161,162^. ALS was first identified in the late nineteenth century by Jean-Martin Charcot, and sufficiently describing the pathogenesis of the disease has since been challenging^163^. Current treatments aim to slow disease development and improve patient quality of life, including riluzole, a glutamatergic release inhibitor, and edaravone, a free radical scavenger that protects against oxidative stress^80,164–166^. Due to the aggressive nature of the condition, with an average prognosis of two to five years postdiagnosis, finding treatments that ameliorate disease progression is of paramount importance^161^.

*SOD1* was the first gene found to be connected to ALS, and subsequent research has linked ~700 genes to ALS risk, including *TARDBP*^161,167,168^, which encodes TDP-43, the accumulation of which is a hallmark of ALS pathology. Nonpathologically, TDP-43 regulates RNA transcription, stability, and splicing^169,170^. When TDP-43 moves from the nucleus to the cytoplasm, it can accumulate and cause neuronal death^171^. In addition, β2AR signaling and components of the GPCR pathway, such as kinase activity, are modified in ALS^122,172,173^. Because of their role in GPCR signal transduction, β-arrestins may play a role in ALS pathogenesis.

Thus far, the role of β-arrestins in ALS is not well established, and research is limited to the therapeutic benefits of targeting β2AR. In vivo, β2AR agonists slow disease progression, as measured by reduced motor neuron loss and delayed motor symptom onset^174–177^. Additionally, ALS shares pathological similarities with FTLD. Namely, TDP-43 accumulation is present in both pathologies^178^. Within FTLD-tau, β-arrestin1 mediates tau accumulation through GPCR signaling^88^. FTLD-tau patients also have increased levels of both β-arrestin1 and β-arrestin2^87,88^. β-arrestins may induce similar β2AR signal transduction in ALS as in FTD. Furthermore, autophagic inhibition by β-arrestin1 and β-arrestin2 oligomers, as seen in the context of FTLD-tau, could also apply to ALS, as autophagy dysfunction is a common hallmark in multiple neurodegenerative diseases^87,88,152,153^. Overlapping aspects of FTD and ALS pathophysiology may provide evidence for the role of β-arrestins within ALS and may identify new therapeutic targets for the condition.

## Discussion

Arrestins are multifunctional proteins that play key roles in receptor desensitization^1,2^, internalization^3–6^, and signaling^1,7,8^ (Fig. 1). While visual arrestins are mostly expressed in the rods and cones of the retina and act on light-sensitive receptors, β-arrestins are ubiquitously expressed and act on a variety of receptors. Although the overall structures of arrestins are similar across the four subtypes, differences in both the N- and C-termini of arrestins differentiate receptor binding affinity between the visual arrestins and β-arrestins^179^. β-arrestin1 and β-arrestin2 share 78% sequence similarity; however, β-arrestin1 is localized to both the cytosol and nucleus, while β-arrestin2 is mostly limited to the cytosol due to its C-terminal NES^27,28^. Furthermore, β-arrestin hetero-oligomers regulate the proper localization of each β-arrestin^84^. Double knockout of both β-arrestin1 and β-arrestin2 is lethal;^9^ however, single knockout of either β-arrestin protein results in a relatively normal phenotype^10,11,87,88^, suggesting that β-arrestin1 and β-arrestin2 can compensate for the loss of the other.

Given that nearly every aspect of metabolism is regulated by the GPCR-β-arrestin axis, it is worth noting that GPCR dysfunction has been implicated in multiple neurodegenerative diseases. While the precise mechanisms by which GPCR dysfunction exacerbates neurodegeneration remain to be elucidated, β-arrestin1 and β-arrestin2 have been implicated in a variety of neurodegenerative diseases. Both β-arrestins are upregulated in the brains of AD^91–93^, PD^133,134^, and FTLD-tau^87,88^ patients compared to normal controls, indicating that β-arrestins are dysregulated in neurodegenerative diseases. However, there is a clear lack of studies investigating the role of β-arrestins in ALS pathogenesis. β-arrestins directly bind to γ-secretase^91,92^, leading to an increase in toxic Aβ production and accumulation in AD (Fig. 2). β-arrestin1 is also upregulated in an MPTP-induced model of PD^133^, and β-arrestins could be a possible therapeutic target for LIDs^144^. The β2AR also regulates *SNCA* gene expression by modifying histone acetylation^131^ (Fig. 4); however, the role of β-arrestins in this mechanism has not yet been explored. Furthermore, β-arrestin oligomers prevent self-interaction of the autophagy cargo receptor p62/SQSTM1^87,88^, resulting in the accumulation of aggregated tau in FTLD-tau (Fig. 3). β-arrestin1 oligomers compete with tau for microtubule binding, leading to destabilization and tau accumulation^88^ (Fig. 3). Given that β-arrestin1 and β-arrestin2 share 78% sequence similarity^1,2^, it is likely that β-arrestin2 is also capable of competing with tau. β-arrestin oligomers are a promising therapeutic target, as cells deficient in oligomers exhibit the same β-arrestin binding capability for clathrin and AP-2^85^, making them indistinguishable from wild-type cells. These studies clearly demonstrate that multiple functions of β-arrestins contribute to neurodegenerative disease pathology, and further studies on β-arrestins are necessary to elucidate disease mechanisms and develop new therapeutic strategies for the prevention or treatment of neurodegeneration.

## References

[1] Ferguson SS (2001). Evolving concepts in G protein-coupled receptor endocytosis: the role in receptor desensitization and signaling. Pharmacol. Rev..

[2] Ferguson SS, Zhang J, Barak LS, Caron MG (1998). Molecular mechanisms of G protein-coupled receptor desensitization and resensitization. Life Sci..

[3] Ferguson SS (1996). Role of beta-arrestin in mediating agonist-promoted G protein-coupled receptor internalization. Science.

[4] Goodman OB (1996). Beta-arrestin acts as a clathrin adaptor in endocytosis of the beta2-adrenergic receptor. Nature.

[5] Shenoy SK, Lefkowitz RJ (2011). beta-Arrestin-mediated receptor trafficking and signal transduction. Trends Pharmacol. Sci..

[6] Zhang J, Ferguson SS, Barak LS, Menard L, Caron MG (1996). Dynamin and beta-arrestin reveal distinct mechanisms for G protein-coupled receptor internalization. J. Biol. Chem..

[7] Lefkowitz RJ (2013). A brief history of G-protein coupled receptors (Nobel Lecture). Angew. Chem. Int. Ed. Engl..

[8] Pitcher JA, Freedman NJ, Lefkowitz RJ (1998). G protein-coupled receptor kinases. Annu. Rev. Biochem..

[9] Zhang M, Liu X, Zhang Y, Zhao J (2010). Loss of betaarrestin1 and betaarrestin2 contributes to pulmonary hypoplasia and neonatal lethality in mice. Dev. Biol..

[10] Bohn LM (1999). Enhanced morphine analgesia in mice lacking beta-arrestin 2. Science.

[11] Conner DA (1997). beta-arrestin1 knockout mice appear normal but demonstrate altered cardiac responses to beta-adrenergic stimulation. Circ. Res..

[12] Feng X, Wang W, Liu J, Liu Y (2011). beta-arestins: multifunctional signaling adaptors in type 2 diabetes. Mol. Biol. Rep..

[13] van Gastel J (2018). beta-arrestin based receptor signaling paradigms: potential therapeutic targets for complex age-related disorders. Front. Pharmacol..

[14] Hirsch JA, Schubert C, Gurevich VV, Sigler PB (1999). The 2.8 A crystal structure of visual arrestin: a model for arrestin’s regulation. Cell.

[15] Hollingsworth JW (2010). Both hematopoietic-derived and non-hematopoietic-derived {beta}-arrestin-2 regulates murine allergic airway disease. Am. J. Respir. Cell Mol. Biol..

[16] Parruti G (1993). Molecular analysis of human beta-arrestin-1: cloning, tissue distribution, and regulation of expression. Identification of two isoforms generated by alternative splicing. J. Biol. Chem..

[17] Radin JN (2005). beta-arrestin 1 participates in platelet-activating factor receptor-mediated endocytosis of Streptococcus pneumoniae. Infect. Immun..

[18] Zhan X, Gimenez LE, Gurevich VV, Spiller BW (2011). Crystal structure of arrestin-3 reveals the basis of the difference in receptor binding between two non-visual subtypes. J. Mol. Biol..

[19] Jiang D, Xie T, Liang J, Noble PW (2013). beta-Arrestins in the immune system. Prog. Mol. Biol. Transl. Sci..

[20] Fan XL, Zhang JS, Zhang XQ, Yue W, Ma L (2003). Differential regulation of beta-arrestin 1 and beta-arrestin 2 gene expression in rat brain by morphine. Neuroscience.

[21] Gurevich EV, Benovic JL, Gurevich VV (2002). Arrestin2 and arrestin3 are differentially expressed in the rat brain during postnatal development. Neuroscience.

[22] Grange-Midroit M (2002). G protein-coupled receptor kinases, beta-arrestin-2 and associated regulatory proteins in the human brain: postmortem changes, effect of age and subcellular distribution. Brain Res. Mol. Brain Res..

[23] Bychkov E, Zurkovsky L, Garret MB, Ahmed MR, Gurevich EV (2012). Distinct cellular and subcellular distributions of G protein-coupled receptor kinase and arrestin isoforms in the striatum. PLoS ONE.

[24] Urs NM (2016). Distinct cortical and striatal actions of a beta-arrestin-biased dopamine D2 receptor ligand reveal unique antipsychotic-like properties. Proc. Natl Acad. Sci. USA.

[25] Tao Y (2015). Astroglial beta-arrestin1-mediated nuclear signaling regulates the expansion of neural precursor cells in adult hippocampus. Sci. Rep..

[26] Hoeppner CZ, Cheng N, Ye RD (2012). Identification of a nuclear localization sequence in beta-arrestin-1 and its functional implications. J. Biol. Chem..

[27] Oakley RH, Laporte SA, Holt JA, Caron MG, Barak LS (2000). Differential affinities of visual arrestin, beta arrestin1, and beta arrestin2 for G protein-coupled receptors delineate two major classes of receptors. J. Biol. Chem..

[28] Wang P, Wu Y, Ge X, Ma L, Pei G (2003). Subcellular localization of beta-arrestins is determined by their intact N domain and the nuclear export signal at the C terminus. J. Biol. Chem..

[29] Scott MG (2002). Differential nucleocytoplasmic shuttling of beta-arrestins. Characterization of a leucine-rich nuclear export signal in beta-arrestin2. J. Biol. Chem..

[30] Gurevich VV, Gurevich EV (2019). GPCR signaling regulation: the role of GRKs and arrestins. Front. Pharmacol..

[31] Gurevich VV (1995). Arrestin interactions with G protein-coupled receptors. Direct binding studies of wild type and mutant arrestins with rhodopsin, beta 2-adrenergic, and m2 muscarinic cholinergic receptors. J. Biol. Chem..

[32] Foord SM (2005). International Union of Pharmacology. XLVI. G protein-coupled receptor list. Pharmacol. Rev..

[33] Ghosh E, Kumari P, Jaiman D, Shukla AK (2015). Methodological advances: the unsung heroes of the GPCR structural revolution. Nat. Rev. Mol. Cell Biol..

[34] Lee Y, Basith S, Choi S (2018). Recent advances in structure-based drug design targeting class A G protein-coupled receptors utilizing crystal structures and computational simulations. J. Med. Chem..

[35] Hu GM, Mai TL, Chen CM (2017). Visualizing the GPCR network: classification and evolution. Sci. Rep..

[36] Ahn S, Nelson CD, Garrison TR, Miller WE, Lefkowitz RJ (2003). Desensitization, internalization, and signaling functions of beta-arrestins demonstrated by RNA interference. Proc. Natl Acad. Sci. USA.

[37] Kohout TA, Lin FS, Perry SJ, Conner DA, Lefkowitz RJ (2001). beta-arrestin 1 and 2 differentially regulate heptahelical receptor signaling and trafficking. Proc. Natl Acad. Sci. USA.

[38] Macey TA, Gurevich VV, Neve KA (2004). Preferential interaction between the dopamine D2 receptor and arrestin2 in neostriatal neurons. Mol. Pharmacol..

[39] Macey TA, Liu Y, Gurevich VV, Neve KA (2005). Dopamine D1 receptor interaction with arrestin3 in neostriatal neurons. J. Neurochem..

[40] Bohn LM, Gainetdinov RR, Lin FT, Lefkowitz RJ, Caron MG (2000). Mu-opioid receptor desensitization by beta-arrestin-2 determines morphine tolerance but not dependence. Nature.

[41] Celver JP, Lowe J, Kovoor A, Gurevich VV, Chavkin C (2001). Threonine 180 is required for G-protein-coupled receptor kinase 3- and beta-arrestin 2-mediated desensitization of the mu-opioid receptor in Xenopus oocytes. J. Biol. Chem..

[42] Dang VC, Chieng B, Azriel Y, Christie MJ (2011). Cellular morphine tolerance produced by betaarrestin-2-dependent impairment of mu-opioid receptor resensitization. J. Neurosci..

[43] Groer CE, Schmid CL, Jaeger AM, Bohn LM (2011). Agonist-directed interactions with specific beta-arrestins determine mu-opioid receptor trafficking, ubiquitination, and dephosphorylation. J. Biol. Chem..

[44] Kovoor A, Celver JP, Wu A, Chavkin C (1998). Agonist induced homologous desensitization of mu-opioid receptors mediated by G protein-coupled receptor kinases is dependent on agonist efficacy. Mol. Pharmacol..

[45] Kovoor A, Nappey V, Kieffer BL, Chavkin C (1997). Mu and delta opioid receptors are differentially desensitized by the coexpression of beta-adrenergic receptor kinase 2 and beta-arrestin 2 in Xenopus oocytes. J. Biol. Chem..

[46] Markova V, Hejnova L, Benda A, Novotny J, Melkes B (2021). beta-arrestin 1 and 2 similarly influence mu-opioid receptor mobility and distinctly modulate adenylyl cyclase activity. Cell Signal..

[47] Pradhan AA (2016). Agonist-specific recruitment of arrestin isoforms differentially modify delta opioid receptor function. J. Neurosci..

[48] Kang J (2005). A nuclear function of beta-arrestin1 in GPCR signaling: regulation of histone acetylation and gene transcription. Cell.

[49] Sanni SJ (2010). beta-arrestin 1 and 2 stabilize the angiotensin II type I receptor in distinct high-affinity conformations. Br. J. Pharmacol..

[50] Jones KT, Echeverry M, Mosser VA, Gates A, Jackson DA (2006). Agonist mediated internalization of M2 mAChR is beta-arrestin-dependent. J. Mol. Signal..

[51] Haider RS (2022). beta-arrestin1 and 2 exhibit distinct phosphorylation-dependent conformations when coupling to the same GPCR in living cells. Nat. Commun..

[52] Luttrell LM, Lefkowitz RJ (2002). The role of beta-arrestins in the termination and transduction of G-protein-coupled receptor signals. J. Cell Sci..

[53] Lefkowitz RJ, Shenoy SK (2005). Transduction of receptor signals by beta-arrestins. Science.

[54] Bouvier M (1988). Removal of phosphorylation sites from the beta 2-adrenergic receptor delays onset of agonist-promoted desensitization. Nature.

[55] Krupnick JG, Goodman OB, Keen JH, Benovic JL (1997). Arrestin/clathrin interaction. Localization of the clathrin binding domain of nonvisual arrestins to the carboxy terminus. J. Biol. Chem..

[56] Laporte SA, Oakley RH, Holt JA, Barak LS, Caron MG (2000). The interaction of beta-arrestin with the AP-2 adaptor is required for the clustering of beta 2-adrenergic receptor into clathrin-coated pits. J. Biol. Chem..

[57] Moo EV, van Senten JR, Brauner-Osborne H, Moller TC (2021). Arrestin-dependent and -independent internalization of G protein-coupled receptors: methods, mechanisms, and implications on cell signaling. Mol. Pharmacol..

[58] Schmid EM (2006). Role of the AP2 beta-appendage hub in recruiting partners for clathrin-coated vesicle assembly. PLoS Biol..

[59] Kang DS (2009). Structure of an arrestin2-clathrin complex reveals a novel clathrin binding domain that modulates receptor trafficking. J. Biol. Chem..

[60] Nobles KN, Guan Z, Xiao K, Oas TG, Lefkowitz RJ (2007). The active conformation of beta-arrestin1: direct evidence for the phosphate sensor in the N-domain and conformational differences in the active states of beta-arrestins1 and -2. J. Biol. Chem..

[61] Xiao K, Shenoy SK, Nobles K, Lefkowitz RJ (2004). Activation-dependent conformational changes in beta-arrestin 2. J. Biol. Chem..

[62] Lin FT, Miller WE, Luttrell LM, Lefkowitz RJ (1999). Feedback regulation of beta-arrestin1 function by extracellular signal-regulated kinases. J. Biol. Chem..

[63] Lin FT (1997). Clathrin-mediated endocytosis of the beta-adrenergic receptor is regulated by phosphorylation/dephosphorylation of beta-arrestin1. J. Biol. Chem..

[64] Shenoy SK, McDonald PH, Kohout TA, Lefkowitz RJ (2001). Regulation of receptor fate by ubiquitination of activated beta 2-adrenergic receptor and beta-arrestin. Science.

[65] Krasel C, Bunemann M, Lorenz K, Lohse MJ (2005). Beta-arrestin binding to the beta2-adrenergic receptor requires both receptor phosphorylation and receptor activation. J. Biol. Chem..

[66] Reiter E, Ahn S, Shukla AK, Lefkowitz RJ (2012). Molecular mechanism of beta-arrestin-biased agonism at seven-transmembrane receptors. Annu. Rev. Pharmacol. Toxicol..

[67] Violin JD, Lefkowitz RJ (2007). Beta-arrestin-biased ligands at seven-transmembrane receptors. Trends Pharmacol. Sci..

[68] Drake MT (2008). beta-arrestin-biased agonism at the beta2-adrenergic receptor. J. Biol. Chem..

[69] Hodavance SY, Gareri C, Torok RD, Rockman HA (2016). G protein-coupled receptor biased agonism. J. Cardiovasc. Pharmacol..

[70] Liu X (2015). beta-arrestin-biased signaling mediates memory reconsolidation. Proc. Natl Acad. Sci. USA.

[71] Allen JA (2011). Discovery of beta-arrestin-biased dopamine D2 ligands for probing signal transduction pathways essential for antipsychotic efficacy. Proc. Natl Acad. Sci. USA.

[72] Chen X (2012). Structure-functional selectivity relationship studies of beta-arrestin-biased dopamine D(2) receptor agonists. J. Med Chem..

[73] Xiao K (2007). Functional specialization of beta-arrestin interactions revealed by proteomic analysis. Proc. Natl Acad. Sci. USA.

[74] Zhuo Y, Gurevich VV, Vishnivetskiy SA, Klug CS, Marchese A (2020). A non-GPCR-binding partner interacts with a novel surface on beta-arrestin1 to mediate GPCR signaling. J. Biol. Chem..

[75] Shukla AK (2010). Arresting a transient receptor potential (TRP) channel: beta-arrestin 1 mediates ubiquitination and functional down-regulation of TRPV4. J. Biol. Chem..

[76] Lefkowitz RJ, Rajagopal K, Whalen EJ (2006). New roles for beta-arrestins in cell signaling: not just for seven-transmembrane receptors. Mol. Cell.

[77] Por ED (2012). beta-arrestin-2 desensitizes the transient receptor potential vanilloid 1 (TRPV1) channel. J. Biol. Chem..

[78] Chen G (2016). beta-arrestin-2 regulates NMDA receptor function in spinal lamina II neurons and duration of persistent pain. Nat. Commun..

[79] Collingridge GL, Peineau S, Howland JG, Wang YT (2010). Long-term depression in the CNS. Nat. Rev. Neurosci..

[80] Fan X, Jin WY, Wang YT (2014). The NMDA receptor complex: a multifunctional machine at the glutamatergic synapse. Front. Cell. Neurosci..

[81] Chen W (2003). Beta-arrestin 2 mediates endocytosis of type III TGF-beta receptor and down-regulation of its signaling. Science.

[82] Perry NA (2019). Arrestin-3 interaction with maternal embryonic leucine-zipper kinase. Cell Signal..

[83] Kovacs JJ (2008). Beta-arrestin-mediated localization of smoothened to the primary cilium. Science.

[84] Storez H (2005). Homo- and hetero-oligomerization of beta-arrestins in living cells. J. Biol. Chem..

[85] Milano SK, Kim YM, Stefano FP, Benovic JL, Brenner C (2006). Nonvisual arrestin oligomerization and cellular localization are regulated by inositol hexakisphosphate binding. J. Biol. Chem..

[86] Boularan C (2007). beta-arrestin 2 oligomerization controls the Mdm2-dependent inhibition of p53. Proc. Natl Acad. Sci. USA.

[87] Woo JA (2020). beta-arrestin2 oligomers impair the clearance of pathological tau and increase tau aggregates. Proc. Natl Acad. Sci. USA.

[88] Woo, J. A. et al. beta-arrestin1 promotes tauopathy by transducing GPCR signaling, disrupting microtubules and autophagy. *Life Sci. Alliance*10.26508/lsa.202101183 (2022). 10.26508/lsa.202101183 PMC8675912 34862271

[89] Hardy J, Selkoe DJ (2002). The amyloid hypothesis of Alzheimer’s disease: progress and problems on the road to therapeutics. Science.

[90] Bossers K (2010). Concerted changes in transcripts in the prefrontal cortex precede neuropathology in Alzheimer’s disease. Brain.

[91] Liu X (2013). β-Arrestin1 regulates γ-secretase complex assembly and modulates amyloid-β pathology. Cell Res..

[92] Thathiah A (2013). β-arrestin 2 regulates Aβ generation and γ-secretase activity in Alzheimer’s disease. Nat. Med..

[93] Liu Y, Liu F, Grundke-Iqbal I, Iqbal K, Gong CX (2011). Deficient brain insulin signalling pathway in Alzheimer’s disease and diabetes. J. Pathol..

[94] Todd NK (2022). GPCR kinases generate an APH1A phosphorylation barcode to regulate amyloid-beta generation. Cell Rep..

[95] Ni Y (2006). Activation of beta2-adrenergic receptor stimulates gamma-secretase activity and accelerates amyloid plaque formation. Nat. Med..

[96] Pasternak SH (2003). Presenilin-1, nicastrin, amyloid precursor protein, and gamma-secretase activity are co-localized in the lysosomal membrane. J. Biol. Chem..

[97] Wang D, Yuen EY, Zhou Y, Yan Z, Xiang YK (2011). Amyloid beta peptide-(1-42) induces internalization and degradation of beta2 adrenergic receptors in prefrontal cortical neurons. J. Biol. Chem..

[98] Thathiah A (2009). The orphan G protein-coupled receptor 3 modulates amyloid-beta peptide generation in neurons. Science.

[99] Nelson CD, Sheng M (2013). Gpr3 stimulates Aβ production via interactions with APP and β-arrestin2. PLoS ONE.

[100] Huang Y (2022). G protein-biased GPR3 signaling ameliorates amyloid pathology in a preclinical Alzheimer’s disease mouse model. Proc. Natl Acad. Sci. USA.

[101] Haque ME, Kim IS, Jakaria M, Akther M, Choi DK (2018). Importance of GPCR-mediated microglial activation in Alzheimer’s disease. Front. Cell Neurosci..

[102] Thathiah A, De Strooper B (2011). The role of G protein-coupled receptors in the pathology of Alzheimer’s disease. Nat. Rev. Neurosci..

[103] Obrenovich ME, Palacios HH, Gasimov E, Leszek J, Aliev G (2009). The GRK2 overexpression is a primary hallmark of mitochondrial lesions during early Alzheimer disease. Cardiovasc. Psychiatry Neurol..

[104] Suo Z, Wu M, Citron BA, Wong GT, Festoff BW (2004). Abnormality of G-protein-coupled receptor kinases at prodromal and early stages of Alzheimer’s disease: an association with early beta-amyloid accumulation. J. Neurosci..

[105] Zhang Y (2014). GRK5 dysfunction accelerates tau hyperphosphorylation in APP (swe) mice through impaired cholinergic activity. Neuroreport.

[106] Suo WZ, Li L (2010). Dysfunction of G protein-coupled receptor kinases in Alzheimer’s disease. ScientificWorldJournal.

[107] Wu H (2020). Abeta monomer induces phosphorylation of tau at Ser-214 through beta2AR-PKA-JNK signaling pathway. FASEB J..

[108] Sharma K (2019). Cholinesterase inhibitors as Alzheimer’s therapeutics (Review). Mol. Med. Rep..

[109] Olivares D (2012). N-methyl D-aspartate (NMDA) receptor antagonists and memantine treatment for Alzheimer’s disease, vascular dementia and Parkinson’s disease. Curr. Alzheimer Res..

[110] Shi M, Chu F, Zhu F, Zhu J (2022). Impact of anti-amyloid-beta monoclonal antibodies on the pathology and clinical profile of Alzheimer’s disease: a focus on aducanumab and lecanemab. Front. Aging Neurosci..

[111] Clinical and neuropathological criteria for frontotemporal dementia. The Lund and Manchester Groups. *J. Neurol. Neurosurg. Psychiatry* **57**, 416–418 (1994). 10.1136/jnnp.57.4.416 PMC1072868 8163988

[112] Arnold SE, Han LY, Clark CM, Grossman M, Trojanowski JQ (2000). Quantitative neurohistological features of frontotemporal degeneration. Neurobiol. Aging.

[113] Cairns NJ (2007). Neuropathologic diagnostic and nosologic criteria for frontotemporal lobar degeneration: consensus of the Consortium for Frontotemporal Lobar Degeneration. Acta Neuropathol..

[114] Tsai RM, Boxer AL (2014). Treatment of frontotemporal dementia. Curr. Treat. Options Neurol..

[115] Beitz JM (2014). Parkinson’s disease: a review. Front Biosci. (Schol. Ed.).

[116] Driver JA, Logroscino G, Gaziano JM, Kurth T (2009). Incidence and remaining lifetime risk of Parkinson disease in advanced age. Neurology.

[117] Dorsey ER (2007). Projected number of people with Parkinson disease in the most populous nations, 2005 through 2030. Neurology.

[118] Pringsheim T, Jette N, Frolkis A, Steeves TD (2014). The prevalence of Parkinson’s disease: a systematic review and meta-analysis. Mov. Disord..

[119] De Virgilio A (2016). Parkinson’s disease: autoimmunity and neuroinflammation. Autoimmun. Rev..

[120] Lerner A, Bagic A (2008). Olfactory pathogenesis of idiopathic Parkinson disease revisited. Mov. Disord..

[121] Goedert M, Spillantini MG, Del Tredici K, Braak H (2013). 100 years of Lewy pathology. Nat. Rev. Neurol..

[122] Azam, S. et al. G-protein-coupled receptors in CNS: a potential therapeutic target for intervention in neurodegenerative disorders and associated cognitive deficits. *Cells*10.3390/cells9020506 (2020). 10.3390/cells9020506 PMC7072884 32102186

[123] Mattila SO, Tuusa JT, Petaja-Repo UE (2016). The Parkinson’s-disease-associated receptor GPR37 undergoes metalloproteinase-mediated N-terminal cleavage and ectodomain shedding. J. Cell Sci..

[124] Gu C (2021). Role of G protein-coupled receptors in microglial activation: implication in Parkinson’s disease. Front. Aging Neurosci..

[125] McGeer PL, Itagaki S, Boyes BE, McGeer EG (1988). Reactive microglia are positive for HLA-DR in the substantia nigra of Parkinson’s and Alzheimer’s disease brains. Neurology.

[126] Fang Y (2021). Opposing functions of beta-arrestin 1 and 2 in Parkinson’s disease via microglia inflammation and Nprl3. Cell Death Differ..

[127] Loniewski K, Shi Y, Pestka J, Parameswaran N (2008). Toll-like receptors differentially regulate GPCR kinases and arrestins in primary macrophages. Mol. Immunol..

[128] Fong AM (2002). Defective lymphocyte chemotaxis in beta-arrestin2- and GRK6-deficient mice. Proc. Natl Acad. Sci. USA.

[129] Wang G (2011). Effects of beta-arrestin 2 on cytokine production of CD4+ T lymphocytes of mice with allergic asthma. Indian J. Exp. Biol..

[130] Volta M (2017). Elevated levels of alpha-synuclein blunt cellular signal transduction downstream of Gq protein-coupled receptors. Cell Signal.

[131] Mittal S (2017). beta2-adrenoreceptor is a regulator of the alpha-synuclein gene driving risk of Parkinson’s disease. Science.

[132] Beaulieu JM (2005). An Akt/beta-arrestin 2/PP2A signaling complex mediates dopaminergic neurotransmission and behavior. Cell.

[133] Bezard E (2005). L-DOPA reverses the MPTP-induced elevation of the arrestin2 and GRK6 expression and enhanced ERK activation in monkey brain. Neurobiol. Dis..

[134] Bychkov ER, Gurevich VV, Joyce JN, Benovic JL, Gurevich EV (2008). Arrestins and two receptor kinases are upregulated in Parkinson’s disease with dementia. Neurobiol. Aging.

[135] Pronin AN, Morris AJ, Surguchov A, Benovic JL (2000). Synucleins are a novel class of substrates for G protein-coupled receptor kinases. J. Biol. Chem..

[136] Tambasco N, Romoli M, Calabresi P (2018). Levodopa in Parkinson’s disease: current status and future developments. Curr. Neuropharmacol..

[137] Lu J, Li X, Wang Q, Pei G (2017). Dopamine D2 receptor and beta-arrestin 2 mediate amyloid-beta elevation induced by anti-parkinson’s disease drugs, levodopa and piribedil, in neuronal cells. PLoS ONE.

[138] Peihua L, Jianqin W (2018). Clinical effects of piribedil in adjuvant treatment of Parkinson’s disease: a meta-analysis. Open Med..

[139] Mizuno Y, Kondo T, Japanese Istradefylline Study G (2013). Adenosine A2A receptor antagonist istradefylline reduces daily OFF time in Parkinson’s disease. Mov. Disord..

[140] Luquin MR, Scipioni O, Vaamonde J, Gershanik O, Obeso JA (1992). Levodopa-induced dyskinesias in Parkinson’s disease: clinical and pharmacological classification. Mov. Disord..

[141] Bargiotas P, Konitsiotis S (2013). Levodopa-induced dyskinesias in Parkinson’s disease: emerging treatments. Neuropsychiatr. Dis. Treat..

[142] Ahlskog JE, Muenter MD (2001). Frequency of levodopa-related dyskinesias and motor fluctuations as estimated from the cumulative literature. Mov. Disord..

[143] Darmopil S, Martin AB, De Diego IR, Ares S, Moratalla R (2009). Genetic inactivation of dopamine D1 but not D2 receptors inhibits L-DOPA-induced dyskinesia and histone activation. Biol. Psychiatry.

[144] Zhang XR (2019). beta-arrestin2 alleviates L-dopa-induced dyskinesia via lower D1R activity in Parkinson’s rats. Aging.

[145] Di Monte DA (2000). Relationship among nigrostriatal denervation, Parkinsonism, and dyskinesias in the MPTP primate model. Mov. Disord..

[146] Shenoy SK, Lefkowitz RJ (2003). Multifaceted roles of beta-arrestins in the regulation of seven-membrane-spanning receptor trafficking and signalling. Biochem J..

[147] Benovic JL, Strasser RH, Caron MG, Lefkowitz RJ (1986). Beta-adrenergic receptor kinase: identification of a novel protein kinase that phosphorylates the agonist-occupied form of the receptor. Proc. Natl Acad. Sci. USA.

[148] Urs NM (2015). Targeting beta-arrestin2 in the treatment of L-DOPA-induced dyskinesia in Parkinson’s disease. Proc. Natl Acad. Sci. USA.

[149] Simola N, Morelli M, Carta AR (2007). The 6-hydroxydopamine model of Parkinson’s disease. Neurotox. Res..

[150] Lim EW (2019). Amyloid-beta and Parkinson’s disease. J. Neurol..

[151] Lu J (2016). An anti-Parkinson’s disease drug via targeting adenosine A2A receptor enhances amyloid-beta generation and gamma-secretase activity. PLoS ONE.

[152] Komatsu M (2006). Loss of autophagy in the central nervous system causes neurodegeneration in mice. Nature.

[153] Nixon RA (2013). The role of autophagy in neurodegenerative disease. Nat. Med..

[154] Alvarez-Erviti L (2010). Chaperone-mediated autophagy markers in Parkinson disease brains. Arch. Neurol..

[155] McNaught KS, Jenner P (2001). Proteasomal function is impaired in substantia nigra in Parkinson’s disease. Neurosci. Lett..

[156] Vogiatzi T, Xilouri M, Vekrellis K, Stefanis L (2008). Wild type alpha-synuclein is degraded by chaperone-mediated autophagy and macroautophagy in neuronal cells. J. Biol. Chem..

[157] Bancher C, Braak H, Fischer P, Jellinger KA (1993). Neuropathological staging of Alzheimer lesions and intellectual status in Alzheimer’s and Parkinson’s disease patients. Neurosci. Lett..

[158] Joachim CL, Morris JH, Kosik KS, Selkoe DJ (1987). Tau antisera recognize neurofibrillary tangles in a range of neurodegenerative disorders. Ann. Neurol..

[159] Lei P (2010). Tau protein: relevance to Parkinson’s disease. Int J. Biochem. Cell Biol..

[160] Singh B (2019). Tau is required for progressive synaptic and memory deficits in a transgenic mouse model of alpha-synucleinopathy. Acta Neuropathol..

[161] Hulisz D (2018). Amyotrophic lateral sclerosis: disease state overview. Am. J. Manag. Care.

[162] Masrori P, Van Damme P (2020). Amyotrophic lateral sclerosis: a clinical review. Eur. J. Neurol..

[163] Goetz CG (2000). Amyotrophic lateral sclerosis: early contributions of Jean-Martin Charcot. Muscle Nerve.

[164] Doble A (1996). The pharmacology and mechanism of action of riluzole. Neurology.

[165] Hubert JP, Delumeau JC, Glowinski J, Premont J, Doble A (1994). Antagonism by riluzole of entry of calcium evoked by NMDA and veratridine in rat cultured granule cells: evidence for a dual mechanism of action. Br. J. Pharmacol..

[166] Writing G, Edaravone ALSSG (2017). Safety and efficacy of edaravone in well defined patients with amyotrophic lateral sclerosis: a randomised, double-blind, placebo-controlled trial. Lancet Neurol..

[167] Rosen DR (1993). Mutations in Cu/Zn superoxide dismutase gene are associated with familial amyotrophic lateral sclerosis. Nature.

[168] Zhang S (2022). Genome-wide identification of the genetic basis of amyotrophic lateral sclerosis. Neuron.

[169] Buratti E (2015). Functional significance of TDP-43 mutations in disease. Adv. Genet..

[170] Morgan S, Orrell RW (2016). Pathogenesis of amyotrophic lateral sclerosis. Br. Med. Bull..

[171] Neumann M (2006). Ubiquitinated TDP-43 in frontotemporal lobar degeneration and amyotrophic lateral sclerosis. Science.

[172] Trieu VN, Liu R, Liu XP, Uckun FM (2000). A specific inhibitor of Janus kinase-3 increases survival in a transgenic mouse model of amyotrophic lateral sclerosis. Biochem. Biophys. Res. Commun..

[173] Guo W, Vandoorne T, Steyaert J, Staats KA, Van Den Bosch L (2020). The multifaceted role of kinases in amyotrophic lateral sclerosis: genetic, pathological and therapeutic implications. Brain.

[174] Paik H (2015). Repurpose terbutaline sulfate for amyotrophic lateral sclerosis using electronic medical records. Sci. Rep..

[175] Teng YD (2006). Therapeutic effects of clenbuterol in a murine model of amyotrophic lateral sclerosis. Neurosci. Lett..

[176] Bartus RT (2016). beta2-Adrenoceptor agonists as novel, safe and potentially effective therapies for amyotrophic lateral sclerosis (ALS). Neurobiol. Dis..

[177] Joassard OR, Durieux AC, Freyssenet D (2013). G. beta2-adrenergic agonists and the treatment of skeletal muscle wasting disorders. Int J. Biochem. Cell Biol..

[178] Hu WT, Grossman M (2009). TDP-43 and frontotemporal dementia. Curr. Neurol. Neurosci. Rep..

[179] Vishnivetskiy SA, Hosey MM, Benovic JL, Gurevich VV (2004). Mapping the arrestin-receptor interface. Structural elements responsible for receptor specificity of arrestin proteins. J. Biol. Chem..

[180] Littmann T (2015). Recruitment of beta-arrestin 1 and 2 to the beta2-adrenoceptor: analysis of 65 ligands. J. Pharmacol. Exp. Ther..

[181] Huang JZ (2018). The delta-opioid receptor and Parkinson’s disease. CNS Neurosci. Ther..

